# Solitary Hepatic Plasmacytoma With IgM Monoclonal Gammopathy: A Case Report

**DOI:** 10.1155/crh/6629709

**Published:** 2025-11-26

**Authors:** Brandon Poppe, Azam Farooqui, Yuan Lin

**Affiliations:** ^1^Internal Medicine Department, Creighton University East Valley Arizona, Chandler, Arizona, USA; ^2^Pathology Department, Chandler Regional Medical Center, Chandler, Arizona, USA

**Keywords:** case report, IgM, liver, plasmacytoma, solitary

## Abstract

Solitary extramedullary plasmacytoma (SEP) is a rare plasma cell neoplasm that typically arises in the upper aerodigestive tract but may occur in other organs, including the liver. IgM-secreting SEPs are exceedingly rare and can be challenging to differentiate from other lymphomas with similar presentations. We report a case of a 73-year-old female who presented with fatigue and cytopenias, later found to have a large hepatic mass. Biopsy revealed a kappa-restricted plasma cell neoplasm with strong CD138, BCL2, CD20, and PAX5 expression and absence of MYD88 L265P mutation via immunohistochemistry. No bone marrow involvement or lymphadenopathy was detected. Although MYD88 was negative, the working diagnosis was initially made as isolated Waldenstrom macroglobulinemia (WM), and the patient was treated as such. Local radiotherapy was contraindicated due to elevated risk with a history of cirrhosis. The patient demonstrated excellent clinical and radiographic response to zanubrutinib therapy by way of improvement in IgM and the size of the liver mass. After reconsideration of the diagnosis, especially in the setting of MYD88 negativity and CD138 positivity, the case seemed to represent an IgM-secreting solitary hepatic plasmacytoma rather than WM. Management did not change when the diagnosis of plasmacytoma was more highly represented due to the radiotherapeutic contraindication and excellent improvement with zanubrutinib. To our knowledge, this represents one of the few documented cases of IgM-secreting solitary hepatic plasmacytoma. This case highlights diagnostic challenges, the importance of immunophenotypic profiling, and the need for individualized management strategies in this rare entity.

## 1. Introduction

Plasmacytomas are rare neoplasms of clonal plasma cells, classified by the World Health Organization (WHO) and International Consensus Classification (ICC) into solitary plasmacytoma of bone (SPB) and solitary extramedullary plasmacytoma (SEP) [1, 2]. SEPs account for approximately 3%–5% of all plasma cell dyscrasias with most cases arising in the head and neck region followed by the gastrointestinal tract. The majority of SEPs do not secrete monoclonal paraproteins, with less than 25% of cases demonstrating detection [3, 4]. Furthermore, primary hepatic plasmacytomas are exceedingly rare, with fewer than 50 reported cases overall and only a handful demonstrating IgM secretion [5–7].

Overall survival (OS) and progression-free survival (PFS) in solitary plasmacytoma depend on location and risk of progression to multiple myeloma (MM). SPBs carry a higher risk of progression (up to 50%–70% at 10 years), while SEPs have a comparatively better prognosis, with 10-year OS around 70% [8, 9]. Hepatic plasmacytomas are so rare that specific OS estimates are not well defined [5, 7].

The risk of distant relapse is higher in SPB than SEP, with a rate of less than 30% in SEP. Of these cases, distant relapse tends to occur within 2-3 years of the initial diagnosis and can often present as SPB or MM [3].

The clinical management of SEPs is primarily local radiotherapy, which is highly effective in controlling disease with the majority of cases becoming cured. Our patient specifically had a diagnosis of cirrhosis and was deemed to be too high risk for local radiotherapy. Systemic therapy may be considered in cases with aggressive and high-risk features, as well as large tumor burden [3, 10].

Zanubrutinib, a Bruton's tyrosine kinase (BTK) inhibitor, has shown efficacy in IgM-driven plasma cell disorders and was used in this case due to the patient's age, comorbidities, and high IgM burden. Multiple Phase 1, 2, and 3 studies, including the ASPEN trial, have demonstrated that zanubrutinib achieves high overall response rates (ORR 85%–95%) and durable PFS in WM patients [11]. Long-term follow-up has also confirmed deep and durable IgM reduction, symptom control, and improved quality of life for these patients [12]. Although zanubrutinib is not approved for the treatment of SEP, our patient demonstrated clinical and radiographic improvement after 2 weeks of therapy.

The key diagnostic challenge lies in distinguishing IgM-secreting plasmacytomas from WM, plasmablastic lymphoma (PBL), and extranodal marginal zone lymphoma (MZL). WM typically requires bone marrow infiltration with lymphoplasmacytic lymphoma and demonstrates MYD88 mutation positivity in > 90% of cases [8, 13]. In contrast, solitary plasmacytomas consist of mature plasma cells, usually lack MYD88 mutations, and exhibit strong CD138 expression without significant lymphoid components [2]. PBL is distinguished by its blastoid morphology, high Ki-67 index, and frequent EBV positivity [14]. MZL shows a predominance of small B-cells with plasma cell differentiation, often with CD20 positivity [15].

Here, we present a case of an IgM-secreting solitary hepatic plasmacytoma that was initially thought to be isolated WM, highlight the diagnostic process, and discuss therapeutic and prognostic considerations in the context of specific patient comorbidities and the existing literature.

## 2. Case Presentation

A 73-year-old female with Type 2 diabetes mellitus, hyperlipidemia, cirrhosis, and osteoporosis presented with progressive fatigue and arthralgias. She denied fever, weight loss, or constitutional symptoms. Laboratory evaluation revealed pancytopenia (hemoglobin 9.1 g/dL, white blood cells 2.8 × 10^9^/L, and platelets 92 × 10^9^/L), normal renal function and liver function tests, and elevated serum immunoglobulins: IgM 3852 mg/dL, IgG 1136 mg/dL, IgA 107 mg/dL, kappa free light chain 1182.5 mg/L, and lambda 10.7 mg/L, with a kappa/lambda ratio of 110.5.

Bone marrow biopsy showed hypercellular marrow with 3% kappa-restricted plasma cells and coexisting ASXL1 and SF3B1 mutations in a myeloid clone. Importantly, MYD88 L265P mutation testing was negative. PET-CT demonstrated a solitary 9.8 × 8.5 × 5.4 cm hypoenhancing lesion in the right hepatic lobe with no evidence of lymphadenopathy or lytic bone lesions. MRI confirmed a solitary hepatic mass.

**Figure 1:**
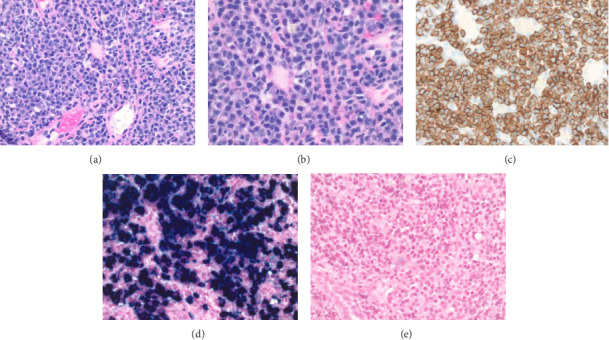
Histopathological images of liver biopsy. (a) H&E low-power field view. (b) H&E high-power field view. (c) IHC stain of CD138. (d) In situ hybridization study for kappa. (e) In situ hybridization study for lambda.

Histopathology of the hepatic lesion revealed a plasma cell neoplasm with strong CD138, CD20, and CD43 positivity, kappa light chain restriction, and absence of significant lymphoid components (Figure 1). Ki-67 proliferation index was 10%–20%. Hepatitis B virus, Hepatitis C virus, and HIV testing were negative at that time as well. Given these findings, the initial working diagnosis became WM which was also discussed and agreed upon with a tertiary academic center. Given the patient's history of cirrhosis, local radiotherapy was deemed too high risk, and she was started on systemic therapy with zanubrutinib 320 mg daily.

Within 2 weeks of starting zanubrutinib, she developed rash, pedal edema, and weakness, prompting temporary discontinuation. During this period, her IgM decreased from 4000 mg/dL to 1400 mg/dL. Imaging demonstrated reduction of the hepatic lesion to 6.3 × 4.4 cm. After steroid support, zanubrutinib was reintroduced at 160 mg daily with good tolerance.

After reevaluation of the working diagnosis, especially in the setting of negative MYD88 and strongly positive CD138, our case seemed to more closely represent an IgM-secreting solitary hepatic plasmacytoma. Although our working diagnosis had changed, we did not feel that a change in management was necessary, especially in the setting of contraindication to local radiotherapy due to elevated risk with cirrhosis as well as clinical and radiographic improvement with zanubrutinib.

She was hospitalized several months later for generalized weakness and was ultimately found to have reactivation of Hepatitis B infection at that time. Zanubrutinib was discontinued, and tenofovir was added. Of note, the patient's EBV IgG testing was positive. She was discharged to an acute rehabilitation facility, and she passed away shortly after.

## 3. Discussion

SEPs are rare entities, representing < 5% of plasma cell dyscrasias [3]. Hepatic SEPs are particularly uncommon, with fewer than 50 cases described and very few associated with IgM secretion [5–7]. In contrast, osseous plasmacytomas are more common but carry a worse prognosis due to higher rates of progression to MM [8, 9]. Reported median OS for SEPs is approximately 8–12 years, whereas SPBs often progress within 5 years [8].

The rarity of hepatic plasmacytomas creates diagnostic uncertainty. In our patient, the absence of MYD88 mutation, lack of marrow infiltration, and presence of mature plasma cell morphology with strong CD138 expression supported plasmacytoma over WM. The differential diagnosis also included PBL, which was excluded due to lack of blastoid morphology, moderate Ki-67, and negative Hepatitis B testing at the time of diagnosis [14]. MZL was unlikely due to the absence of small B-cell aggregates [15].

Therapeutically, localized plasmacytomas are generally treated with radiotherapy, achieving > 90% local control [10]. However, systemic therapy may be indicated in bulky and high-risk disease [3, 10]. Our patient was treated with zanubrutinib, a BTK inhibitor, which resulted in significant IgM reduction and tumor shrinkage. While not standard of care for plasmacytomas, its use highlights a tailored approach in IgM-driven variants with large tumor burden, especially when conventional therapy may be less suitable as in our patient with a history of cirrhosis.

The prognosis of hepatic plasmacytomas is not well characterized and is even more difficult to compare to similar SEPs at other sites, given our patient had an IgM monoclonal gammopathy [7]. Nevertheless, patients with IgM-secreting plasmacytomas may require closer monitoring for systemic progression given their clinical overlap with WM.

This case underscores the importance of comprehensive immunophenotypic profiling in unusual plasma cell neoplasms. By integrating morphology, immunohistochemistry, molecular testing, and clinical presentation, we established a diagnosis of solitary IgM-secreting hepatic plasmacytoma. Our findings emphasize the need for reporting additional cases to clarify the prognosis and optimize therapy in this rare entity.

## 4. Conclusion

IgM-secreting SEP of the liver is an exceptionally rare diagnosis that poses significant diagnostic challenges due to its overlap with WM and other lymphoid neoplasms. Careful integration of histopathology, immunophenotypic markers, and molecular testing is essential for accurate classification. Our patient demonstrated both clinical and radiographic response to targeted therapy with zanubrutinib, highlighting the potential role of systemic treatment in selected cases where radiotherapy alone may not be optimal. This case contributes to the limited body of literature on hepatic plasmacytomas and emphasizes the importance of case reporting to guide future diagnostic and therapeutic strategies in this rare plasma cell disorder.

## Data Availability

The authors have nothing to report.

## References

[1] (2022). *WHO Classification of Haematolymphoid Tumours*.

[2] Campo E., Jaffe E., Cook J. (2022). The International Consensus Classification of Mature Lymphoid Neoplasms: A Report From the Clinical Advisory Committee. *Blood*.

[3] Soutar R., Lucraft H., Jackson G. (2004). Guidelines on the Diagnosis and Management of Solitary Plasmacytoma of Bone and Solitary Extramedullary Plasmacytoma. *British Journal of Haematology*.

[4] Kilciksiz S., Karakoyun-Celik O., Agaoglu F., Haydaroglu A. (2012). A Review for Solitary Plasmacytoma of Bone and Extramedullary Plasmacytoma. *The Scientific World Journal*.

[5] Alexiou C., Kau R., Dietzfelbinger H. (1999). Extramedullary Plasmacytoma: Tumor Occurrence and Therapeutic Concepts. *Cancer*.

[6] Chalopin T., Barillot I., Biny J. (2017). Primary Solitary Plasmacytoma of the Liver-Successful Treatment with Fractionated Stereotactic Radiotherapy (Cyberknife®): A Case Report. *Journal of Medical Case Reports*.

[7] Shao Y., Zhang H., Zhang Q., Yan S., Wang W. (2019). Primary Solitary Extramedullary Plasmacytoma of the Liver Mimicking Hepatocellular Carcinoma: Case Report. *International Journal of Clinical and Experimental Medicine*.

[8] Dimopoulos M., Kiamouris C., Moulopoulos L. (1999). Solitary Plasmacytoma of Bone and Extramedullary Plasmacytoma. *Hematology-Oncology Clinics of North America*.

[9] Katodritou E., Kastritis E., Dalampira D. (2024). Characteristics, Outcome, and Prognostic Factors for Survival and Progression to Multiple Myeloma of Solitary Plasmacytomas: A 30-Year Experience of the Greek Myeloma Study Group. *Blood*.

[10] Tsang R., Gospodarowicz M., Pintilie M. (2001). Solitary Plasmacytoma Treated With Radiotherapy: Impact of Tumor Size on Outcome. *International Journal of Radiation Oncology, Biology, Physics*.

[11] Tam C., Garcia-Sanz R., Opat S. (2022). Aspen: Long-Term Follow-Up Results of a Phase 3 Randomized Trial of Zanubrutinib (ZANU) Versus Ibrutinib (IBR) in Patients With Waldenström Macroglobulinemia (WM). *Journal of Clinical Oncology*.

[12] Trotman J., Opat S., Gottlieb D. (2020). Zanubrutinib for the Treatment of Patients With Waldenström Macroglobulinemia: 3 Years of Follow-Up. *Blood*.

[13] Owen R., Treon S., Al-Katib A. (2003). Clinicopathological Definition of Waldenstrom’s Macroglobulinemia: Consensus Panel Recommendations from the Second International Workshop on Waldenstrom’s Macroglobulinemia. *Seminars in Oncology*.

[14] Castillo J. J., Reagan J. L. (2011). Plasmablastic Lymphoma: A Systematic Review. *The Scientific World Journal*.

[15] Isaacson P. G. (1999). Mucosa-Associated Lymphoid Tissue Lymphoma. *Seminars in Hematology*.

